# Serine ether glycerophospholipids: Decrements in the frontal cortex associated with mild cognitive impairment and Alzheimer’s disease

**DOI:** 10.3389/fnagi.2022.981868

**Published:** 2022-08-08

**Authors:** Paul L. Wood, Randall L. Woltjer

**Affiliations:** ^1^Metabolomics Unit, College of Veterinary Medicine, Lincoln Memorial University, Harrogate, TN, United States; ^2^Department of Pathology, Oregon Health and Science University, Portland, OR, United States

**Keywords:** serine ether glycerophospholipid, frontal cortex, MCI, Alzheimer’s, dementia

## Abstract

Ether glycerophospholipids (GPL) are involved in membrane fluidity and fusion. Vinyl-ether GPL are also conjectured to provide antioxidant capacity in the brain. The roles of these lipids in the processes involved in the development of dementia are not understood but choline and ethanolamine vinyl-ether GPL (i.e., plasmalogens) are decreased in the brains of subjects with dementia. In contrast, serine ether and vinyl-ether GPL have not been investigated in human brain. We therefore undertook an evaluation of these lipids, utilizing high-resolution mass spectrometry (HR-MS), in tissues from control and dementia subjects that we had previously characterized in-depth. We can report for the first time that a number of serine ether GPL and a more limited number of serine plasmalogens are present in human frontal cortex. In addition, we found that some of these frontal cortex lipids are decreased in Mild Cognitive Impairment (MCI), early-onset Alzheimer’s disease (EOAD), and late-onset AD (LOAD). In contrast no alterations in serine ether GPL were monitored in the frontal cortex of donors with schizophrenia, demonstrating disease specificity. These data suggest that further studies of the roles of ether GPL, including serine ether GPL, in brain function are worthy of undertaking.

## Introduction

Ether glycerophospholipids (GPL), including plasmalogens, are unique membrane lipids that contribute to membrane fluidity and are conjectured to provide antioxidant capacity at the membrane surface (Wood, 2012). Plasmalogens are ether GPL characterized by a vinyl-ether linkage at sn-1 of the glycerol backbone. The biosynthesis (Wood, 2012) of ether GPL first involves the addition of a fatty alcohol at sn-1, in peroxisomes. In the case of plasmalogens this is followed by desaturation at the carbon-carbon bond adjacent to the ether linkage (Gallego-García et al., 2019; Werner et al., 2020), a reaction that is catalyzed *via* an endoplasmic reticular oxidoreductase (EC 1.14.19.77). The sn-3 polar headgroups of ether lipids and plasmalogens are mainly phosphocholine and phosphoethanolamine. Both of these GPL families have been shown to have variable levels of decrements in the neocortex of subjects with dementia (Wood, 2012), possibly resulting from peroxisomal dysfunction (Kou et al., 2011). However, serine ether GPL and plasmalogens remain to be investigated in the human brain.

**FIGURE 1:**
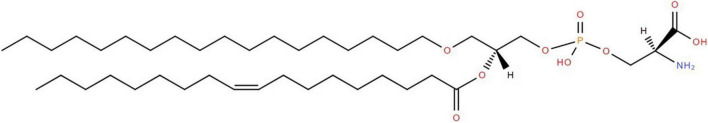
Structure of PSe 36:1 (e18:0/18:1). The glycerol backbone possesses phosphoserine as the polar headgroup at sn-3, oleic acid *via* an acyl bond at sn-2, and stearyl alcohol *via* an alkyl bond (i.e., ether) at sn-1. In the case of a plasmalogen the bond at sn-1 would be an alkenyl bond (i.e., a double bond between the first 2 carbons after the -O-CH_2_.

Serine ether GPL (PSe; i.e., alkyl-acyl serine GPL; Figure 1) and serine plasmalogens (PSp; i.e., alkenyl-acyl serine GPL), have been reported from studies of human retina and optic nerve (Nagy et al., 2012), human lens (Deeley et al., 2008, 2009; Ellis et al., 2010), rat prostate (Pulido et al., 1986), the mouse macrophage cell line RAW 264.7 (Ivanova et al., 2010), gills of bivalves (Kraffe et al., 2004), the fungus pneumocystis carinii (Kaneshiro et al., 1998), a number of bacteria (van Golde et al., 1973; Oe et al., 2008; Rezanka et al., 2011), murine mamillary tumors (Sekar et al., 2022), and coral (Imbs and Velansky, 2021).

In studies of human retina and optic nerve, PSp 36:1 was present in both tissues while PSp 39:3 and 41:3 were detected exclusively in the retina (Nagy et al., 2012). The ether lipids PSe 36:1 and 38:4 also were only detected in the retina. In the case of the human lens ether serine GPLs predominate and are localized as a ring in the outermost boundaries of the lens (Deeley et al., 2008). These studies suggest that ether and vinyl-ether serine GPL play unique and specialized functions in the human ocular system.

With this background, we focused on the examination of these lipids within the human brain to evaluate if any alterations in serine ether GPL levels could be detected in dementia.

## Materials and methods

### Dementia clinical samples

De-personalized post-mortem tissues (frontal cortex gray and white matter) were provided to the Oregon Brain Bank by volunteer subjects who were evaluated at Oregon Health and Science University (IRB 00001623). These are the same samples utilized in a prior study (Sekar et al., 2022) where donors were categorized by age and level of cognitive impairment as assessed in the Layton Aging and Alzheimer’s Disease Center with annual neurological and neuropsychological evaluations, and clinical dementia rating (CDR) as assigned by an experienced neurologist. Controls had normal examinations. The clinical diagnosis of dementia was determined by clinical team consensus conference. Patients with dementia met the National Institute for Neurological and Communicative Disorders and Stroke-Alzheimer’s Disease and Related Disorder Association diagnostic criteria for clinical AD, and had a CDR > 1. All subjects had pathologic diagnoses determined at the Oregon Alzheimer’s Disease Center. Tissue acquisition and use followed institutional review board requirements. Frontal lobe tissue was flash frozen and stored at −80°C for biochemical studies described here. Brain tissue was evaluated by a neuropathologist for features of neurodegenerative disease, which were identified and staged as previously reported (Erten-Lyons et al., 2013) and according to the National Alzheimer’s Coordinating Center (NACC)’s uniform data collection. A demographics overview of the donors is presented in Table 1 and more detailed information is available in our previous publication (Wood et al., 2015).

**TABLE 1:** Demographics of the donors providing the post-mortem frontal cortex samples for our analyses of dementia tissues.

Diagnosis	Age ± SD	Age range	*N*	Gender (M/F)
Controls	67.3 ± 11.8	49–82	20	11/9
MCI	90.5 ± 7.9	57–79	19	4/15
EOAD (<85)	75.5 ± 6.9	58–84	17	9/8
LOAD (>85)	90.5 ± 4.6	86–101	17	6/11

MCI, Mild Cognitive Impairment; EOAD, early-onset Alzheimer’s disease; LOAD, late-onset AD.

### Schizophrenia clinical samples

Frontal cortex brain samples were provided by the University of California, Los Angeles (UCLA) brain bank and are the same tissues as reported in a previous publication (Wood, 2014; Wood et al., 2014). Schizophrenia patients were diagnosed based on the Structural Interview for Diagnostic and Statistical Manual of Mental Disorders IV (DSM-IV). The demographics of the donors are presented in Table 2 and more detailed information is available in our previous publications (Wood, 2014; Wood et al., 2014).

**TABLE 2:** Demographics of the donors providing the post-mortem frontal cortex samples for our analyses of schizophrenia.

Diagnosis	Age ± SD	Age range	*N*	Gender (M/F)
Controls	61.8 ± 17.1	33–85	10	6/4
Schizophrenia	72.1 ± 17.4	35–92	10	4/6

### Sample processing

Frontal cortex tissues (40–80 mg) were mixed with 1 mL of water and 1 mL of methanol (Wood, 2017). The samples were sonicated prior to the addition of 2 mL of methyl-tert-butyl ether (MTBE). Subsequently the tubes were vigorously shaken at room temperature for 30 min., followed by centrifugation at 4,000 × *g* for 30 min. One milliliter of the upper organic layer was dried by centrifugal vacuum evaporation and dissolved in 150 μL of isopropanol: methanol: chloroform (4:2:1) containing 15 mM ammonium acetate for mass spectrometry (MS) analysis.

### High-resolution mass spectrometry analyses of seine ether glycerophospholipids

Electrospray ionization mass spectrometric (ESI-MS) analysis (3.2 kV, capillary temp. of 320°C, sheath gas of 10) of frontal cortex extracts utilized high-resolution (140,000 at 200 amu) data acquisition, with millimass accuracy (<2 ppm mass error) on an orbitrap mass spectrometer (Thermo Q Exactive). Samples were infused for 30 s in positive ion mode followed by 30 s in negative ion mode at 20 μL/min. Infusions were followed by successive 500 μL washes of the infusion line with methanol and hexane/ethyl acetate (3:2) to minimize ghost effects. The observed ions and their peak intensities were transferred to an Excel spreadsheet containing the calculated cations for phosphatidylcholine (PC) 34:2 and PCp 38:4 and the calculated anions for PEp 36:2, serine ether GPL, and serine plasmalogens. Only lipids with <2 ppm mass error are reported here.

For the structural validation of serine ether and vinyl-ether GPL we utilized MS/MS. Prior publications have demonstrated that both ether (Pulido et al., 1986; Nagy et al., 2012) and vinyl-ether (Nagy et al., 2012) serine GPL generate the associated ether and vinyl-ether phosphatidic acid product ions (PAe and PAp, respectfully) resulting from the loss of serine. These PAs further fragment to generate the associated lysophosphatidic acids of ether PA (LPAe) and vinyl-ether PA (LPAp). In the case of PSp, MS/MS generates the unique alkenyl anion associated with the sn-1 vinyl-ether substitution (Nagy et al., 2012), while the sn-2 fatty acids are product anions of PSe (Kraffe et al., 2004). This knowledge base provided the basis for us to generate the data required to validate serine ether lipids in human frontal cortex (Supplementary material). All masses were queried in the Lipid Maps database, at <2 ppm mass accuracy, and found to be unique to serine alkyl and alkenyl GPL.

### Data analyses

To provide data that allowed a comparison of the relative abundance of serine ether lipids relative to other lipids we utilized phosphatidylcholine 34:2 as our reference lipid, similar to utilizing a housekeeping gene in PCR analysis. PC 34:2 is very stable and levels of this GPL were not altered in our previous studies of the frontal cortex in dementia (Wood et al., 2015) or schizophrenia (Wood, 2014; Wood et al., 2014). Data are therefore presented as a% of the associated PC 34:2 peak intensity (i.e., ratio of the PSe or PSp peak intensity to the peak intensity of PC 34:2 presented as a%). The levels of PC34:2, based on [^2^H_31_] PC 34:1 as the internal standard, were 0.23 and 0.19 nmoles per mg wet weight for gray matter (GM) and white matter (WM) respectfully.

This calculation automatically corrects for differences in tissue weights. PC 34:2 is a valid choice as a stable baseline comparator since we have shown previously that it is unaltered (i.e., stable) in dementia which associated with brain shrinkage (Wood et al., 2014) and in schizophrenia where there is no brain shrinkage (Wood, 2014; Wood et al., 2014). It is worthy to note that the tissues used in this study are from the same brain slices as used in our previous studies (Wood, 2014; Wood et al., 2014, 2015). The sample sizes were too small to detect and age effects on lipid levels as previously noted in our lipidomics studies of these same tissues (Wood, 2014; Wood et al., 2014, 2015).

Data are presented in bar graphs as mean ± SEM. Data were analyzed by 1-way ANOVA, followed by the Tukey–Kramer test to determine differences between groups.

## Results

### High-resolution mass spectrometry analyses of neocortical serine ether and vinyl-ether glycerophospholipids

**FIGURE 2:**
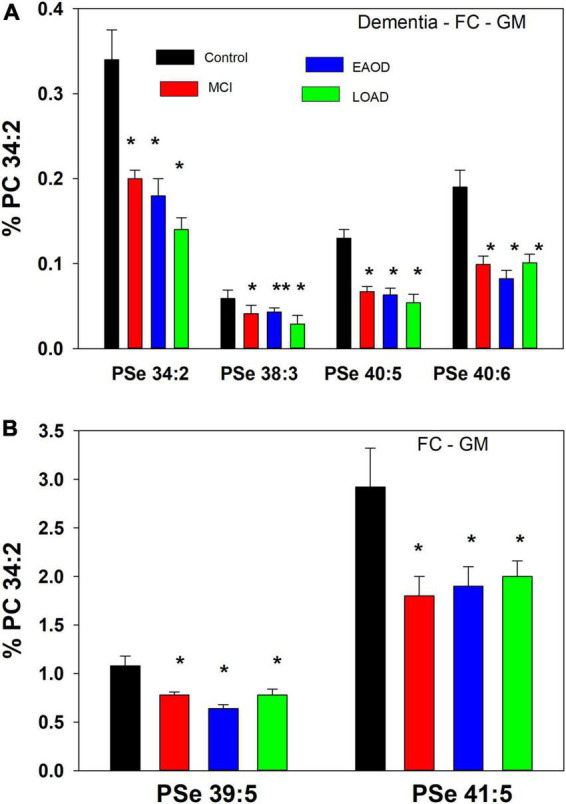
**(A)** Relative levels of ether (PSe) serine GPL in the frontal cortex gray matter (GM) in controls (black bar), Mild Cognitive Impairment (MCI; red bars), early onset dementia (EOAD; blue bars) and late-onset dementia (LOAD; green bars). *p < 0.01; ^**^p < 0.05. Relative levels are the ratio of the peak intensity of each serine lipid to that of the peak intensity of phosphatidylcholine 34:2 [phosphatidylcholine (PC) 34:2] multiplied by 100 to yield a percentage (Mean ± SEM). **(B)** Relative levels of ether (PSe) and vinyl-ether (PSp) serine GPL in the frontal cortex white matter (GM) in controls (black bar), Mild Cognitive Impairment (MCI; red bars), early onset dementia (EOAD; blue bars) and late-onset dementia (LOAD; green bars). *p < 0.01. Relative levels are the ratio of the peak intensity of each serine lipid to that of the peak intensity of phosphatidylcholine 34:2 (PC 34:2) multiplied by 100 to yield a percentage (Mean ± SEM).

Based on prior publications we scanned for serine ether (PSe) and vinyl-ether (PSp) GPL anions in the range of 740– 850 amu, utilizing electrospray ionization coupled with high-resolution mass spectrometry (HR-MS; 140,000 resolution; <2 ppm mass error). MS/MS of both ether (Pulido et al., 1986; Nagy et al., 2012) and vinyl-ether (Werner et al., 2020) serine GPL generates the associated ether and vinyl-ether phosphatidic acid product ions (PAe and PAp, respectfully), resulting from the loss of serine. These PAs further fragment to generate the associated LPAe and vinyl-ether PA (LPAp) that result from loss of the sn-2 fatty acid. In human frontal cortex GM we monitored serine ether GPL but no serine plasmalogens (Figure 2).

In the case of PSp, MS/MS also generates the alkenyl anion associated with the sn-1 vinyl-ether substitution (Nagy et al., 2012). In contrast, sn-2 fatty acids are product anions of PSe (Pulido et al., 1986). With this knowledge base, we determined, based on the detection of the 18:1 alkenyl anion (Supplementary material), that human frontal cortex white matter contains two PSp species, namely PSp 36:1 (p18:1/18:0) and PSp 36:2 (p18:1/18:1; Figure 3). Also associated with the PSp 36:1 mass were two PSe 36:2 species and in the case of PSp 36:2 two PSe 36:3 species (Supplementary material). In contrast to the restricted number of PSp species, we monitored eight other PSe species for which the structural validations are presented in Supplementary material. For the even-carbon PSe species, the associated sn-2 fatty acid substituents were robust product signals with MS/MS analyses. However, in the case of odd-carbon PSe species we did not monitor the associated sn-2 fatty acids with MS/MS analyses, despite monitoring the associated LPAe. These data therefore limit our identifications of odd-carbon PSe species as tentative since analytical standards are not available to validate our observations.

### Ether and vinyl-ether serine glycerophospholipids in the frontal cortex in donors with dementia

The lipid extracts from frontal cortex gray (GM) contained only serine ether GPL and no serine plasmalogens. These included PSe 34:2, 38:3, 40:5, 40:6, 39:5, and 41:5, which in all cases were decreased in MCI, EOAD, and late-onset AD (LOAD) donors (Figure 2). The even-carbon lipids were validated by MS/MS with generation of the associated phosphatidic acid (PA) (i.e., loss of the serine headgroup), LPA (i.e., loss of the sn-2 fatty acid from the PA), and sn-2 fatty acid products (Supplementary material). In the case of the odd-carbon lipids, we monitored the associated PA and LPA products but not the sn-2 fatty acids, making their identification tentative.

**FIGURE 3:**
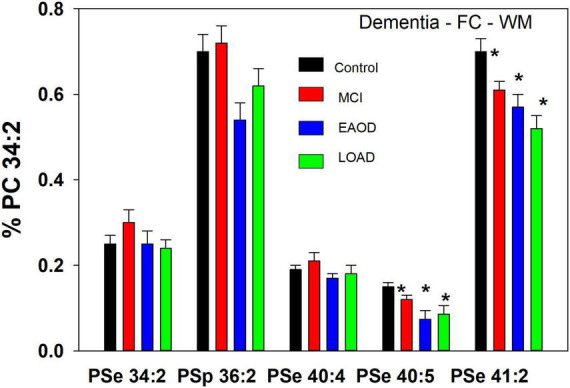
Relative levels of ether (PSe) serine GPL in the frontal cortex white matter (WM) in controls (white bar) and subjects with a history of schizophrenia (gray bars). Relative levels are the ratio of the peak intensity of each serine lipid to that of the peak intensity of phosphatidylcholine 34:2 (PC 34:2) multiplied by 100 to yield a percentage (Mean ± SEM).

In a comparison to the relative levels of choline and ethanolamine plasmalogens in frontal cortex GM (Table 3), even-carbon serine ether GPL are present at much lower levels (Figure 2). In contrast, odd-carbon serine ether GPL (Figure 2) are present at levels, in the range of choline plasmalogens, based solely on signal intensities since analytical standards are not available for absolute quantitation (Table 3).

**TABLE 3:** Relative levels (peak intensities as a% of phosphatidylcholine (PC) 34:2 peak intensity) of choline plasmalogen 38:4 (PCp 38:4) and ethanolamine plasmalogen 36:2 (PEp 36:2) in control human frontal cortex. Mean ± SEM (*N* = 20).

Control tissue	PCp 38:4 (% of PC 34:2)	PEp 36:2 (% of PC 34:2)
GM	5.9 ± 0.36	34.9 ± 4.1
WM	12.1 ± 0.75	52.7 ± 2.2

In WM, a different profile of serine ether GPL was monitored. In this case PSe 34:2 and 40:4 were monitored and the relative levels of these ether lipids were not altered in dementia donors (Figure 3). In contrast, PSe 40:5 and 41:2 were decreased in MCI, EOAD, and LOAD donors. Also, in WM we monitored the serine plasmalogen 36:2 which was decreased in MCI, EOAD, and LOAD donors (Figure 3). The presence of this serine plasmalogen in WM clearly distinguishes the lipid profile of this central nervous system (CNS) compartment from the GM.

In a comparison to the relative levels of choline and ethanolamine plasmalogens in frontal cortex WM (Table 3), the serine ether GPL are present at trace levels (Figure 3).

### Ether serine glycerophospholipids in frontal cortex gray matter in donors with schizophrenia

**FIGURE 4:**
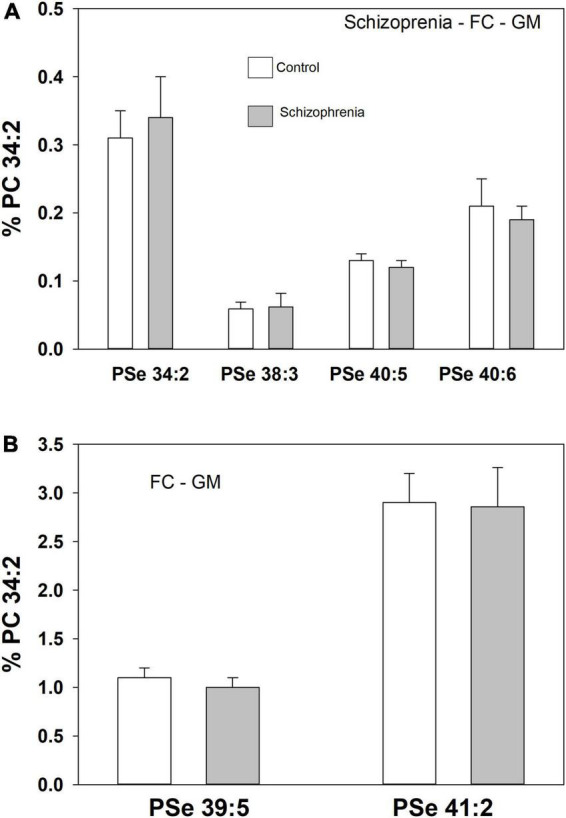
Relative levels of ether (PSe) serine GPL in the frontal cortex gray matter (GM) in controls (white bar) and subjects with a history of schizophrenia (gray bars). Relative levels are the ratio of the peak intensity of each serine lipid to that of the peak intensity of phosphatidylcholine 34:2 (PC 34:2) multiplied by 100 to yield a percentage (Mean ± SEM).

A number of PSe species also were monitored in the frontal cortex GM of both controls and donors with schizophrenia. In this case there were no differences in the relative levels of these ether GPL (Figure 4).

## Discussion

Previous studies of ether GPL in Alzheimer’s disease have reported variable decreases in ethanolamine plasmalogens in the cortex (Ginsberg et al., 1995; Han et al., 2001; Kou et al., 2011; Chan et al., 2012; Wood et al., 2015) potentially as a result of peroxisomal dysfunction (Gallego-García et al., 2019). In our previous studies of the GM and WM tissues used in this study we monitored decrements in ethanolamine plasmalogens in the frontal cortex of EOAD and LOAD donors but not in MCI (Wood et al., 2015). In this study, we did not detect PSp in the cortical GM while monitoring a number of PSe species, all of which were decreased in MCI, EOAD, and LOAD donors. The majority of these ether lipids were at trace levels relative to choline and ethanolamine plasmalogens, except for PSe 39:5 and 41:5 which are at similar abundance levels to PCp 38:4. In WM PSe 40:5 and 41:2 also demonstrated decreased tissue levels while PSe 34:2, PSe 40:4, and PSp36:2 were unaltered. In all cases these ether lipids were present at trace levels. The roles of these ether lipids in CNS function remain to be defined but based on the observations that ethanolamine and choline ether lipids are involved in membrane fluidity and fusion (Brites and Wanders, 2004), it is interesting to speculate that serine ether lipids may be involved in these specialized functions also. In addition, the observations that serine ether lipids are confined to specific regions of the human lens (Ellis et al., 2010), further suggests that they potentially participate in specialized functions. The potential role of decrements in serine ether GPL in the pathophysiology of dementia is not understood at this time. However, the decrements observed in MCI subjects could indicate a potential role in dysfunctional synaptic transmission early in the disease process.

Our data demonstrating decrements in serine ether GPL also lends further support to the contention that there is decreased peroxisomal function in AD (Kou et al., 2011). The full significance of altered peroxisomal function requires more examination since many complex biochemical processes are conducted by these unique organelles. At the least, the alteration in ether lipids has the potential to alter membrane fluidity and the function of enzymes and receptors present in lipid rafts (Wood, 2012). In contrast choline and ethanolamine ether GPL (Wood, 2012), the low levels of most serine ether GPL makes it unlikely that they are a significant source of sn-2 fatty acids for signal transduction and biochemical pathways.

To evaluate the specificity of the changes we monitored in serine ether lipids in dementia, we also evaluated the frontal cortex GM from donors with schizophrenia. In the same tissue samples utilized in this study, we previously monitored increased levels of both ethanolamine and choline plasmalogens (Wood et al., 2014) and N-acylphosphatidyl serines (i.e., N-acyl-diacyl GPS) (Wood, 2014). In this study of the same tissues none of the serine ether GPL levels were altered. These data demonstrate that serine ether GPL are altered in two different cognitive disorders, namely MCI and AD, but not in schizophrenia, a psychiatric disorder. The similar decreases in these lipids in MCI and AD suggest that they are not related to the deposition of plaques and tangles since the densities of these protein deposits are significantly greater in AD donors. Furthermore, since not all MCI cases progress to Alzheimer’s disease (Chan et al., 2012; Michaud et al., 2017), our data suggest that changes in serine ether GPL occur early in the disease process since subjects with only mild cognitive effects have low levels of these lipids.

## Data availability statement

The raw data supporting the conclusions of this article will be made available by the authors, without undue reservation.

## Ethics statement

The studies involving human participants were reviewed and approved by the Oregon Health and Science University (IRB 00001623). The patients/participants provided their written informed consent to participate in this study.

## Author contributions

Both authors listed have made a substantial, direct, and intellectual contribution to the work, and approved it for publication.
