# Supplementary material for: Serine ether glycerophospholipids: Decrements in the frontal cortex associated with mild cognitive impairment and Alzheimer’s disease
Source: Front Aging Neurosci. 2022 Aug 8;14:981868. doi: 10.3389/fnagi.2022.981868 (PMC9393623; doi:10.3389/fnagi.2022.981868)
Supplement: Supplementary file 1 [file Data_Sheet_1.PDF]

Supplementary Material

Supplementary Table 1: Structural elucidation of serine plasmalogen (PSp) and serine ether GPL (PSe) species extracted from human frontal cortex.

Lipid	Anion	ppm	[PAp/e]-	ppm	LPA	Anion	ppm	sn-2 FA	ppm
PSe 34:2	744.5186	0.65	657.4865	0.92					
PS (e16:1/18:1)					e16:1	393.2412	0.85	18:1	1.1
PS (e18:2/16:0)					e18:2	401.2462$	0.93	16:0	0.79
PSp 36:1/PSe 36:2	772.5498	0.78	685.5178	0.85					
PS (p18:1/18:0)*					p18:1	401.2462$	0.93		
PS (e16:2/16:0)					e16:2	391.2255	0.67	16:0	0.89
PS (e18:1/18:1)					e18:1	403.2619$	0.89	18:1	0.79
PSp 36:2/PSe 36:3	770.5432	0.73	683.5021	0.88					
PS (p18:1/18:1)*					p18:1	419.2568	0.85		
PS (e18:1/18:2)					e18:1	403.2619$	0.77	18:2	0.97
PS (e18:3/18:0)					e18:3	417.2412	0.64	18:0	0.85
PSe 38:3	798.5655	0.64	711.5334	0.84					
PS (e18:2/20:1)					E18:2	419.2568	0.71	20:1	0.89

PSe 39:2	814.5968	0.89	727.5647	0.92					
PS (e18:2/21:2)					e18:2	419.2568	0.77		
PSe 39:5	808.5498	0.77	721.5178	0.46					
PS (e18:2/21:3)					e18:2	419.2568	0.88		
PSe 40:4	824.5811	0.86	737.5491	0.81					
PS (e18:0/22:4)					e18:0	405.2775$^{\$}$	0.84	22:4	0.12
PSe 40:5	822.5655	0.92	735.5334	1.0					
PS (e18:1/22:4)					e18:1	403.2619$^{\$}$	0.93	22:4	0.70
PS (e18:2/22:3)					e18:2	419.2568	0.82	22:3	1.1
PSe 40:6	820.5498	0.84	733.5178	0.89					
PS (e18:2/22:4)					e18:2	419.2568	0.99	22:4	0.99
PS (e18:0/22:6)					e18:0	405.2775$^{\$}$	0.89	22:6	0.82
PSe 41:2	842.6381	0.95	755.5960	0.91					
PS (e18:2/23:0)					e18:2	419.2568	0.86		
PSe 41:3	840.6124	0.88	753.5804	0.85					

PS (e18:2/23:1)					e18:2	419.2568	0.85			
PSE 41:5	836.5811	0.92	749.5491	0.96						
PS (e18:2/23:3)					e18:2	419.2568	0.79			

*, 18:1 alkenyl product 265.2537 (0.85 ppm) was detected with MS/MS; $, -$H_2O$ (note: the hydrated species were also detected but at lower peak intensities). Anion, calculated anion; FA, fatty acid; LPA, lysophosphatidic acid [M-H-serine-sn2 FA]$^-$; PA, phosphatidic acid [M-H-serine]$^-$; ppm, parts-per-million mass error for observed anion.

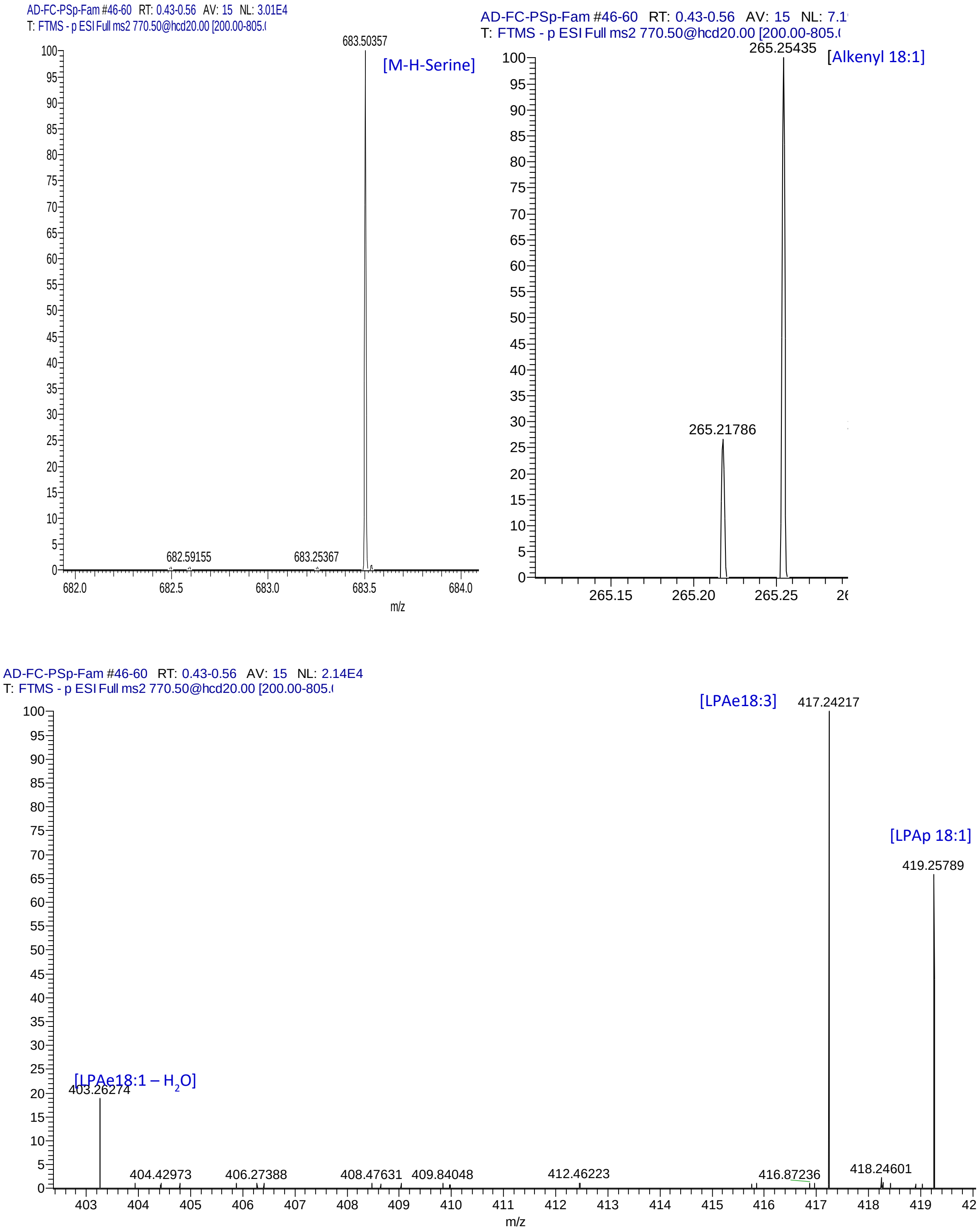

Supplementary Fig. 1. MS/MS spectrum of 770.5 which is composed of PSp 36:2 (PS p18:1/18:1), PSe 36:3 (PS e18:3/18:0), and PSe36:3 (PS e18:1/18:2).
